# Supplementary material for: Coronatine is more potent than jasmonates in regulating Arabidopsis circadian clock
Source: Sci Rep. 2020 Jul 30;10:12862. doi: 10.1038/s41598-020-69627-2 (PMC7393363; doi:10.1038/s41598-020-69627-2)
Supplement: Supplementary file 2 — Supplementary Information 2. [file 41598_2020_69627_MOESM2_ESM.docx]

**Supplemental Figure Legends**

**Figure S1. COR is required for *P. syringae* virulence in LL.** (A) Bacterial growth. (B) Images to show disease symptoms. 25-d-old plants grown in LD were transferred to LL for 1 d followed by spraying with *P. syringae* 25 h after onset of LL. Statistical analysis was performed with One-way ANOVA with post-hoc Tukey HSD test (asterisks for P<0.001).

**Figure S2. Expression of selected circadian genes with MJ or COR treatment.** (A) MJ treatment. (B) COR treatment. Gene expression plots were generated by ggplot2 package in R using time-series RNA-seq data from MJ (A) or COR (B) treated samples vs. mock treated samples [18, 19].

**Figure S3.** **Heatmap analysis of expression of defense genes with MJ or COR treated samples**. (A) Gene expression heatmap of MJ-treated samples. (B) Gene expression heatmap of COR-treated samples. Differential gene expression values in (A) and (B) were obtained from a comparison with mock treated samples [18, 19] followed by a Log_2_ transformation.

**Figure S4. Gene expression analysis by qRT-PCR.** 7 d old LD-entrained seedlings were transferred to LL for 1 d and then treated with MJ (100 µM) or COR (10 µM) for gene expression analysis by qRT-PCR. Water was included as a mock treatment. Expression of CCA and LUX with MJ treatment was shown previously [5]. These experiments were repeated two times with similar results.

**Figure S5. Seedling growth inhibition assays.** (A) Relative seedling leaf area with COR treatment. (B) Relative seedling leaf area with MJ treatment. (C) Relative seedling leaf area with JA-Ile treatment. At the end of luminescence recording, seedlings were photographed and measured for leaf area with ImageJ. The average leaf area of water-treated samples of each genotype was set to 1 and used to calculate the relative leaf area of chemical-treated seedlings of the same genotype. Data represent mean ± SEM (n=12). Statistical analysis was performed by One-way ANOVA post-hoc Tukey HSD test. Different letters indicate significant difference among the samples treated at the same time point (P<0.05). These experiments were repeated three times with similar results.

**Figure S6. JA-Ile treatment affects clock activity** LD-entrained 5 d old seedlings were transferred to LL for 1 d and were treated with JA-Ile at 25 h (top of each panel) or 37 h (bottom of each panel). Luminescence was recorded at 1-h intervals for five days and analyzed for clock activity. A1-D1 Expression of *TOC1:LUC* in Col-0. A2-D2 Expression of *PRR7:LUC* in Col-0. A1-A2 Luminescence traces. RLU: Relative luminescence units. The color indicates JA-Ile concentration, black for 0, magenta for 10 µM, and gray for 100 µM. B1-B2 Normalized amplitude. The amplitude of the reporter was normalized to the relative leaf area shown in Figure S5. C1-C2 Period. D1-D2 Phase shift. Data represent mean ± SEM (n=12). Statistical analysis was performed by One-way ANOVA post-hoc Tukey HSD test. Different letters indicate significant difference among the samples (P<0.05). These experiments were repeated three times with similar results.
